# From Peer-Reviewed to Peer-Reproduced in Scholarly Publishing: The Complementary Roles of Data Models and Workflows in Bioinformatics

**DOI:** 10.1371/journal.pone.0127612

**Published:** 2015-07-08

**Authors:** Alejandra González-Beltrán, Peter Li, Jun Zhao, Maria Susana Avila-Garcia, Marco Roos, Mark Thompson, Eelke van der Horst, Rajaram Kaliyaperumal, Ruibang Luo, Tin-Lap Lee, Tak-wah Lam, Scott C. Edmunds, Susanna-Assunta Sansone, Philippe Rocca-Serra

**Affiliations:** 1 Oxford e-Research Centre, University of Oxford, 7 Keble Road, OX1 3QG, United Kingdom; 2 GigaScience, BGI HK Research Institute, 16 Dai Fu Street, Tai Po Industrial Estate, Hong Kong, People’s Republic of China; 3 InfoLab21, Lancaster University, Bailrigg, Lancaster, LA1 4WA, United Kingdom; 4 Nuffield Department of Medicine, Experimental Medicine Division, John Radcliffe Hospital, Headley Way, Headington, Oxford, OX3 9DU, United Kingdom; 5 Department of Human Genetics, Leiden University Medical Center, P.O. Box 9600, 2300 RC Leiden, The Netherlands; 6 HKU-BGI Bioinformatics Algorithms and Core Technology Research Laboratory & Department of Computer Science, University of Hong Kong, Pokfulam, Hong Kong, People’s Republic of China; 7 School of Biomedical Sciences and CUHK-BGI Innovation Institute of Trans-omics, The Chinese University of Hong Kong, Shatin, Hong Kong, People’s Republic of China; University of Illinois-Chicago, UNITED STATES

## Abstract

**Motivation:**

Reproducing the results from a scientific paper can be challenging due to the absence of data and the computational tools required for their analysis. In addition, details relating to the procedures used to obtain the published results can be difficult to discern due to the use of natural language when reporting how experiments have been performed. The Investigation/Study/Assay (ISA), Nanopublications (NP), and Research Objects (RO) models are conceptual data modelling frameworks that can structure such information from scientific papers. Computational workflow platforms can also be used to reproduce analyses of data in a principled manner. We assessed the extent by which ISA, NP, and RO models, together with the Galaxy workflow system, can capture the experimental processes and reproduce the findings of a previously published paper reporting on the development of SOAPdenovo2, a *de novo* genome assembler.

**Results:**

Executable workflows were developed using Galaxy, which reproduced results that were consistent with the published findings. A structured representation of the information in the SOAPdenovo2 paper was produced by combining the use of ISA, NP, and RO models. By structuring the information in the published paper using these data and scientific workflow modelling frameworks, it was possible to explicitly declare elements of experimental design, variables, and findings. The models served as guides in the curation of scientific information and this led to the identification of inconsistencies in the original published paper, thereby allowing its authors to publish corrections in the form of an errata.

**Availability:**

SOAPdenovo2 scripts, data, and results are available through the GigaScience Database: http://dx.doi.org/10.5524/100044; the workflows are available from GigaGalaxy: http://galaxy.cbiit.cuhk.edu.hk; and the representations using the ISA, NP, and RO models are available through the SOAPdenovo2 case study website http://isa-tools.github.io/soapdenovo2/. **Contact**: philippe.rocca-serra@oerc.ox.ac.uk and susanna-assunta.sansone@oerc.ox.ac.uk.

## Introduction

Several reports have highlighted the practical difficulties in reproducing results from published experiments [1–4]. That a basic tenet of scientific research cannot be fulfilled has fuelled growing concerns from stakeholders with an acute interest in scientific reproducibility such as universities, industry, funding agencies, the wider research community as well as the public. A failure to reproduce published scientific findings adversely affects scientific productivity and, in worse cases, may lead to retraction [5]. Moreover, it casts doubt on the quality of the peer-review process. Therefore, the stakeholders of scholarly communication have renewed efforts to mitigate the shortcomings of scientific reporting. For instance, amongst the incentives tried by publishers are the lift on restrictions on the length of methods sections, the creation of data publication platforms, such as GigaScience [6] and Scientific Data [7], the provision of a statistical review of numerical results where appropriate and the requirement for data to be deposited in open-access repositories. These efforts have in part been driven by position statements from funding agencies, publishers and researchers advocating more widespread data sharing [8–10]. Research Communities such as the Research Data Alliance [11] or the Force11 [12] have in fact spearheaded efforts aimed at changing the state of affair of scholarly digital communication. Both groups have issued recommendations and called for active participation and development of new models and practices. The central role of funding agencies can not be forgotten. In fact, the NIH program Big Data to Knowledge (BD2K) constitutes a major initiative, aimed at making data dissemination and data preservation for all NIH funded work a reality, by mandating the creation of data access plans for all new grant applications [13].

Computational frameworks and data models now exist which can be used to structure scientific data and their analyses. In this article, we investigate three conceptual community data models for providing structured reporting of findings and scientific workflows for capturing the data analysis pipeline. Investigation/Study/Assay (ISA) is a widely used, general-purpose, metadata tracking framework with an associated suite of open-source software, delivering rich descriptions of the experimental condition information [14]. The ‘Investigation’ provides the project context for a ‘Study’ (a research question), which itself contains one or more types of ‘Assays’ (taking analytical measurements and key data processing and analysis steps). The transformations of data underlying an analysis can be represented as steps within a scientific workflow that can be automatically executed and repeated on platforms such as Taverna [15] and Galaxy [16]. Nanopublication (NP) is a model which enables specific scientific assertions to be annotated with supporting evidence, published and cited [17]. Lastly, the Research Object (RO) model enables the aggregation of the digital resources contributing to findings of computational research, including results, data and software, as citable compound digital objects [18]. Combined, these conceptual models facilitate the validation of the findings and assist the reuse and understanding of the results.

Our study addresses the question of whether such data and workflow representation frameworks can be used to assist in the peer review process, by facilitating evaluation of the accuracy of the information provided by scientific articles with respect to their repeatability. We applied the ISA framework, the Galaxy workflow platform, NP and RO models on an article in GigaScience. Jointly published by BioMed Central and BGI, GigaScience is linked to a database, GigaDB [19], hosting large scale datasets, but also scripts used to analyse a dataset associated with the publications. The article [20] was selected on the basis that all the data, the analysis scripts used and extensive documentation were all publicly available in GigaDB [21]. However, as we will show, even deposition of the data and the software required to perform the analysis in an open repository does not guarantee reproducibility. Even though seven referees had tested a number of the data sets and analysis scripts [22], we found issues with reproducing the actual results published in the article. In this paper, we show how the combination of data and workflow representation models play a crucial part in highlighting important experimental elements, otherwise easily missed, and enhance data reporting, data review and data publication processes.

## Results

### SOAPdenovo2 experiment overview

The article by Luo *et al* [20] describes the development of SOAPdenovo2 and its evaluation as a computational tool for the *de novo* assembly of genomes from small DNA segments read by next generation sequencing (NGS). Improvements were made at each step of the de Bruijn graph based algorithm implemented by SOAPdenovo1. This new algorithm was evaluated against four NGS data sets from two bacterial genomes (*S. aureus* and *R. sphaeroides*), one insect genome (*B. impatiens*) from the Genome Assembly Gold-standard Evaluations (GAGE competition [23, 24]), and the human YH Asian Genome data set [25]. The performance of SOAPdenovo2 was compared with its predecessor, SOAPdenovo1 [26], and ALL-PATHS-LG [27].

### Reproducing the results from the paper with Galaxy workflows

Our reproducibility effort focused on developing Galaxy workflows, re-creating the data analysis processes used in calculating the results presented in Tables 2, 3 and 4 of the original manuscript [20], which show the performance of SOAPdenovo2 in assembling the four genomes aforementioned. Prior to developing the workflows, SOAPdenovo2, its pre- and post-processing tools had to be integrated into a Galaxy server [28] using their command-line interfaces. These were then combined within Galaxy workflows, thus recapitulating the computational steps the SOAPdenovo2 authors used in *bash* and *perl* scripts for assembling the genomes and evaluate the performance of their new assembler [21]. Due to both insect and human data sets’ large sizes, we were not able to develop executable workflows for assembling these genomes as our public server could not meet the memory needs of up to 155 GB, as indicated by the SOAPdenovo2 authors for building the human genome.

Galaxy workflows were developed to assemble the genomes for *S. aureus* and *R. sphaeroides*. However, for those genomes, two additional steps, not found in the authors’ *bash* scripts, were required to reproduce the statistics. A step was needed to break scaffolds between any gaps into separate sequences. Another was needed to calculate the actual genome assembly statistics in original Table 2 from [20], performed by an analysis script [29] developed for use in the GAGE genome assembly competition [24]. Both of these steps were added to the SOAPdenovo2 genome assembly Galaxy workflows for *S. aureus* and *R. sphaeroides*. The results obtained from the execution of these workflows were almost identical to those published in [20] and are available in Table 1 of the present manuscript. By deploying SOAPdenovo1 and ALL-PATHS-LG [30] as tools within Galaxy, it was possible to re-implement genome assembly and reproduce the results from [20], albeit with minor discrepancies (see Table 1, present manuscript).

**Table 1:** Results from reproducing Table 2 of the original paper, where the original results are shown in between parenthesis.

**Species**	**Algorithm**			**Contig**				**Scaffold**	
		**Number**	**N50(kb)**	**Errors**	**N50 corrected (kb)**	**Number**	**N50(kb)**	**Errors**	**N50 corrected (kb)**
S. aureus	SOAPdenovo1	79	148.6	156	23	49	342	0	342
	SOAPdenovo2	80	98.6	25	71.5	38	1086	2	1078
	ALLPATHS-LG	37	149.7	13	***119 (117.6)***	***11***	1477	1	1093
R. sphaeroides	SOAPdenovo1	***2241 (2242)***	3.5	***400 (392)***	2.8	956	***106(105)***	***24(18)***	***68 (70)***
	SOAPdenovo2	721	18	106	14.1	333	2549	4	2540
	ALLPATHS-LG	190	41.9	***30(31)***	36.7	32	3191	0	***0 (3310)***

**Table 2:** Predictor and response variables for the SOAPdenovo2 study, as identified in the ISA-TAB documents.

**Variable Type**	**Variable Name**	**Variable Values**
**Predictor Variables**	genome assembly algorithm (OBI:0001522)	ALLPATH-LG
		SOAPdenovo1
		SOAPdenovo2
	genome size (PATO:0000117)	small
		medium
		large
**Response Variables**	genome coverage	
	computation run time	
	memory consumption	

### Modeling the experimental process using ISA

The ISA research object provides constructs to describe study design and experimental variables. It can accommodate minimal information guidelines [31], which may insist on reporting such information. When approaching the work by [20], we applied some of the same curation rules implemented by Metabolights, Toxbank and Stem Cell Commons projects to describe key information about the overall study design. The basic principles are, first, to identify predictor and variables, and then, assess the replication levels in order to build a very synthetic yet accurate picture of the experimental design. For instance, in toxicogenomics, guidelines for performing experiments in animals are well documented and establish regulations to limit animal use and animal suffering. OECD guidelines 408 [32] for repeated dose toxicity studies detail how to list perturbators, the intensity of the perturbation as well as its duration. The guidelines also provide advice on biological replications and on how to minimize animal use while retaining statistical power. The availability of this knowledge served as a basis for establishing consistent data collection and assessment procedure for the reporting of *in-vivo* treatment based studies in fairly generic ways, ISA model allowing the represent independent variable using the Study Factor Name declaration and Factor Value field to report the actual factor levels. In fact, most intervention studies can be handled in a similar fashion. Hence, the methodology was straightforward to follow, even in a field remote to Toxicology as the study of efficiency of computational methods. In fact, applying those principles led to the rapid identification and recovery of key information. As indicated in the experiment overview, in [20], four genomes from three distinct phyla, representing 3 points along a genome size gradient covering several orders of magnitude, were used to test 3 genome assembly software. Thus, we summarized the experiment as a 3 × 3 factorial design, with two independent variables or factors declared: software and genome size—cast as Study Factor Name in the ISA syntax. For both variables, three discrete levels were found and reported in a Factor Value field in an ISA assay table. As [20] compares *de novo assembler* methods, the independent variable levels do not affect the samples and do not need to be reported at the study table level.

Next, we represented the data points, or members of each study group. As [20] accounts for refinements to the first published diploid genome sequence of an Asian individual (referred to here as *Chinese Han genome* or *YH genome*) [25, 33] with new reads generated on the newer Illumina platform, the assay template “*genome sequencing using nucleotide sequencing*” was chosen from the various wet lab workflow templates available from ISAcreator, the curation tool in the ISA infrastructure. This ensures meeting annotation requirements covering key steps of specific experimental processes, enabling direct deposition to the European Nucleotide Archive [34] or to the Short Read Archive repository [35] using ISAcreator format interconversion function. This representation allows distinguishing newly generated data from downloaded data when declaring inputs in the genome assembly processes.

The ISA model minimal implementation guidelines instruct to systematically report data file and software locations as resolvable identifiers. The guideline resulted in detecting missing files (for an example, refer to S1 Table of the supplementary material of the present manuscript and unresolvable file references). It also revealed a lack of unambiguous identification of the reference genomes used to perform the alignment step. We achieved a resolution through direct communication with the authors of [20], clarifying that the NCBI human reference genome *hg19* mentioned in [20], known to GenBank as “Genome Reference Consortium Human Build 37 (GRCh37)” [36], corresponds to GenBank Assembly *ID:GCA_000001405.1*. This fact allowed to disambiguate the reference genome version from its subsequent releases (7 in total).

We then focused on identifying the response variables, and their units, used to assess assembly software efficiency. Information from result tables in [20] was extracted, identifying six metrics: i.) genome coverage (as a percentage), ii.) contig N50, iii.) scaffold N50 (stated in kb or base pairs (bp)), iv.) number of errors, v.) run time (stated in hours) and vi.) peak memory usage (stated in gigabytes). For each response variable, S2 Table of the supplementary material, collates definitions as reported in [27]. The first four metrics provide estimates on assembly efficiency and accuracy, whilst the last two give insights into computational efficiency and therefore depict the savings the most efficient computational method can offer in terms of time and memory. Correspondence with the authors confirmed that all metrics were calculated using an analysis script from GAGE [24], executed in a fixed environment on each of the genome assembly software output files, thus guaranteeing protocol consistency. Using ISA, sequence analysis and software comparison outputs were reported relying on Derived Data File fields used to supply file paths or Gigascience document object identifiers (DOIs) to relevant objects.

**Fig 1:**
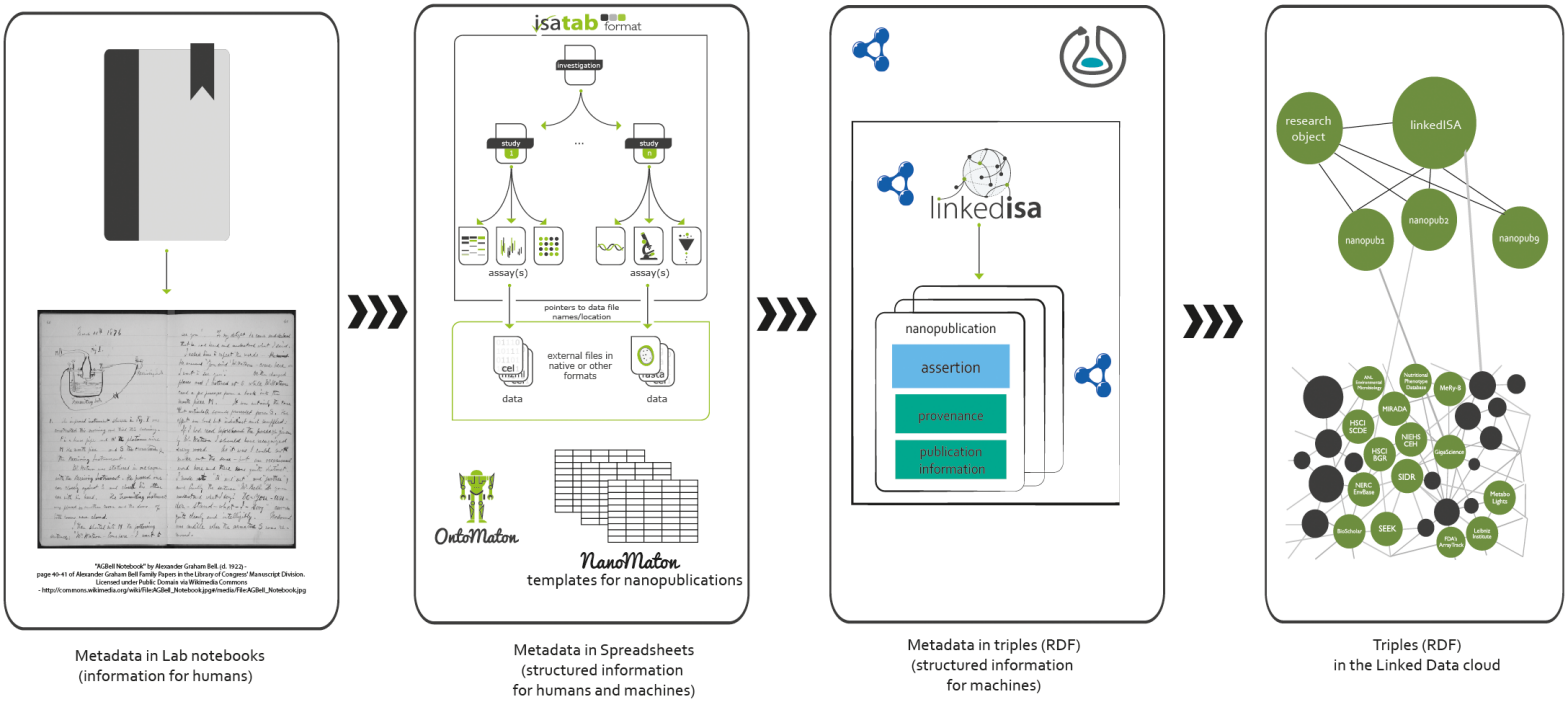
A graphical representation showing the role of ISA, Nanopublication and Research model in progressively structuring experimental information, moving from hand written notes in laboratory books to semi-structure tab-delimited files and fully explicit linked data.

The ISA representation of the study by [20] is released as an ISA-Tab archive and a semantic representation using the Resource Description Framework (RDF) [37]. The latter relies on the *linkedISA* software component [38], using a mapping to Open Biological and Biomedical Ontologies (OBO) resources [39]. In particular, mapping to the Ontology for Biomedical Investigations (OBI) [40] ensures interoperability with several projects using OBI and alignment with ISA configurations. Fig 1 provides an overview of the process of structuring information from laboratory books to digital archives. In addition, linkedISA can also be configured with additional mappings and for the conversion of the SOAPdenovo2 experiment, we used a mapping to the provenance ontology (PROV-O) [41].

### Publishing findings as Nanopublications

First, we considered following key findings to be expressed as nanopublications:

1. genome coverage increased and memory consumption was 2/3 lower (during the point of largest memory consumption) over the human data when comparing SOAPdenovo2 against SOAPdenovo1.
2. improvements in contig and scaffold N50 metrics when considering SOAPdenovo2 versus SOAPdenovo1 for *S. aureus*, *R. sphaeroides* and YH dataset, as presented in Tables 2 and 4 of [20].

These key findings were extracted from the abstract and main conclusions of the article. We tracked the provenance of the statements by identifying the corresponding rows in the tables of the article [20] and complemented them with more statements extracted from those tables, taking into account the response variables, as identified earlier. This process resulted in 9 assertions, which were turned into 9 nanopublications.

**Fig 2:**
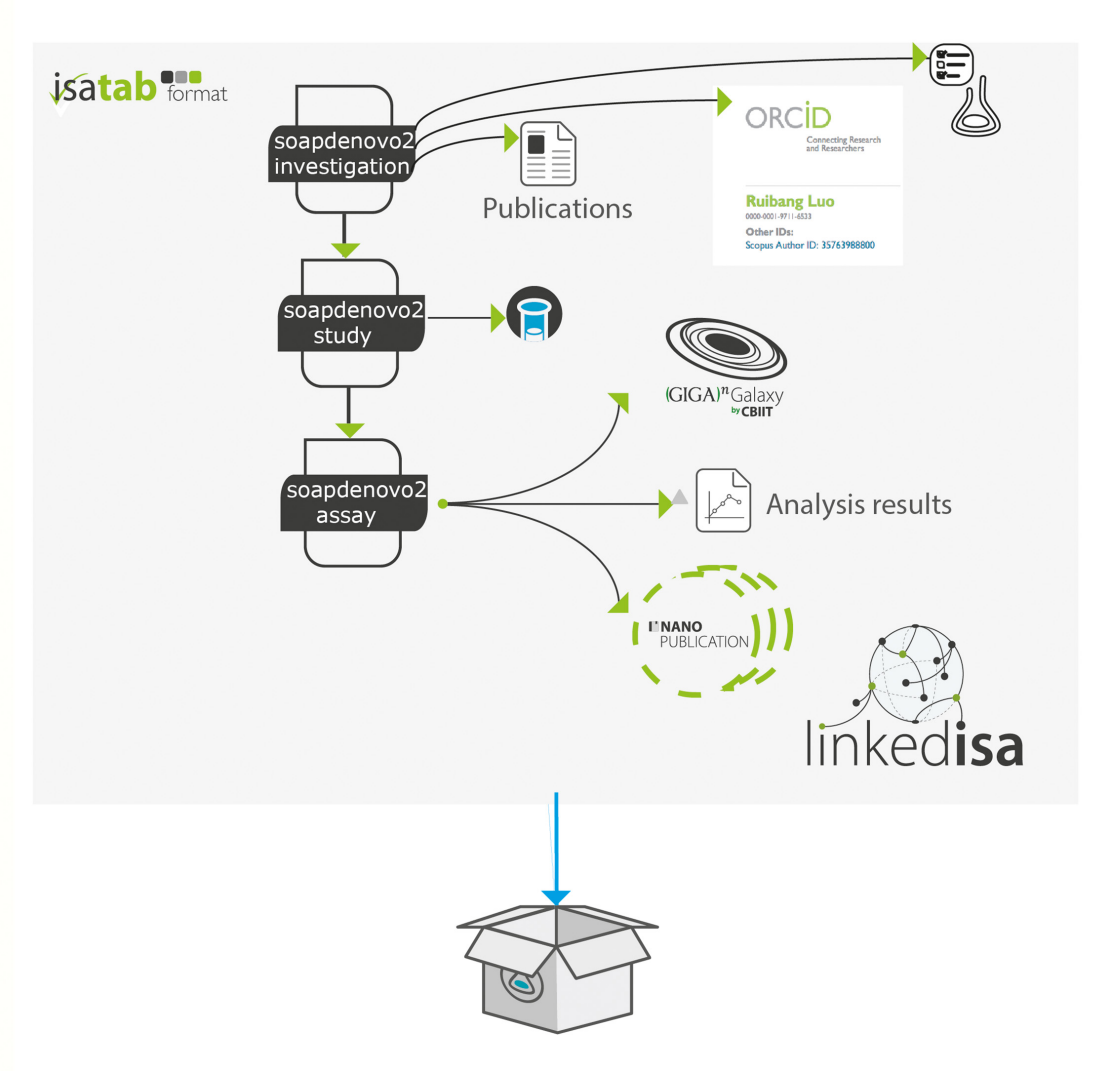
Another view of the complementary aspects of these research object models, highlighting the reliance of persistent identifiers (such as ORCID), and references to Galaxy workflows hosted on GigaScience Servers.

**Fig 3:**
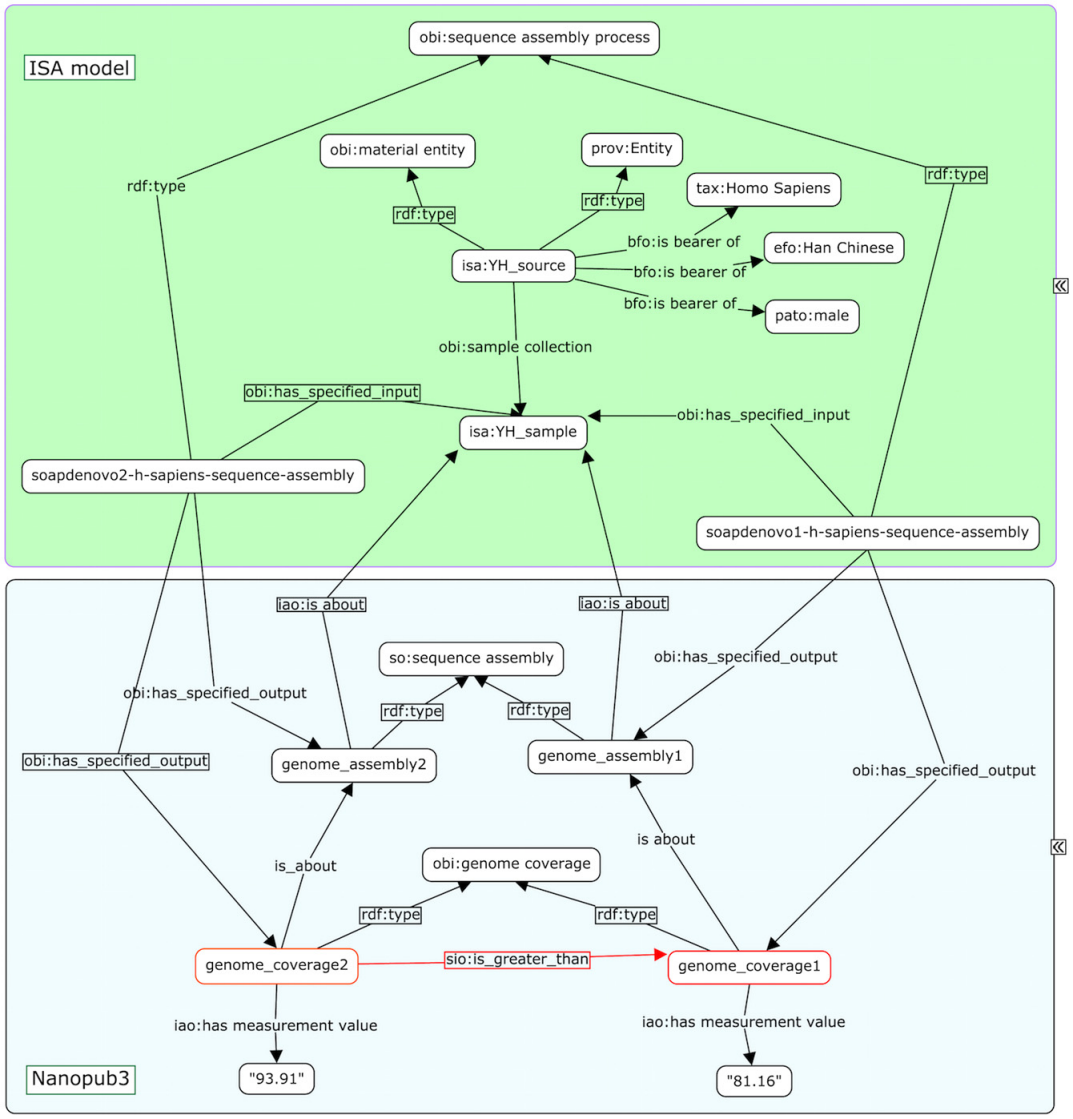
A detailed view showing how linked data representation of an ISA experiment (upper pane, in green background), with one of the finding expressed as Nanopublication statements, (lower pane), where a red outline indicates the key statement.

The nanopublications were created following a novel methodology that combined OntoMaton [42] and NanoMaton [43] software tools. Collected statements were structured as triples in a Google spreadsheet, using the OntoMaton widget, a component of the ISA software suite [42] that accesses community ontologies portals [44, 45]. The collaborative environment allowed review, discussion and incremental improvement until satisfactory expressivity and clarity was reached. The statements were processed with the NanoMaton software component, which converted the OntoMaton templates to RDF. A conceptual overview of how ISA and a nanopublication are related is presented in Fig 1 and Fig 2, while a detailed view, rendered as a graph, is available in Fig 3.

The nanopublication guidelines advocate the use of existing semantic types to create nanopublications as Linked Data. We thus relied on OBI [40], STATistics Ontology (STATO) [46] and SIO [47] but none provided semantic types for response variables such as *genome coverage*, *random access memory*, *computation runtime* that were used in [20]. Gaps in domain coverage is a known caveat in the Semantic Web approach, especially when carrying out *de-novo* semantic modelling. It either requires filing a term request in existing resources or creating a new ontology. We chose the former, owing to familiarity with OBI procedures, thus ensuring rapid processing and the completion of nanopublications seamlessly consistent with the linkedISA RDF representation. The terms submitted to OBI through this work have been made available since March 2014 relying on partial import from Sequence Ontology and direct additions.

The provenance component of the NP model also required special attention. Since the NP translates a written statement from a manuscript into RDF triples, it inherently retains an interpretative aspect by those formulating it. We therefore included all contributing parties, from the original authors [20] to the *semantic translators* who crafted the NPs.

### Preserving computational workflows and aggregating ISA and NP representation as Research Objects

The ISA framework establishes scientific rigour by requesting scientists to report their experiment results along with information about the data analysis process and experiment design. The Research Object model [18] advocates the sharing of information related to a study as one structured aggregation object, in order to facilitate the validation of the findings and assist the reuse and understanding of the results. Our previous study has shown a need to preserve additional material along with experiment results in order to enhance their re-usability and reproducibility [48]. This understanding has also been confirmed by other recent studies on preservation best practices [49, 50]. Therefore, in the process of applying RO models, this understanding is reflected as a set of 5 minimal principles, namely, 1) ensuring that the data inputs used in an experiment are made available as part of the RO; 2) ensuring that basic metadata is associated with an RO and its components, including how they relate to each other, so that they can be interpreted; 3) ensuring that basic provenance information is associated with the RO and its components, like where they came from, when *etc.*, to assist attribution, versioning, citation, and reproducibility; 4) wherever applicable, ensuring that evolution of individual component of an RO and the RO itself can be tracked; and finally 5) ensuring that all these material are aggregated components of an RO, as identifiable objects, so that they can be referred to, shared, and cited. In this study, these principles were applied to capture resources related to the Galaxy workflows created by GigaScience for generating the results presented in Table 2 in [20]. The RO thus contains links to the input data used by the workflow, the Galaxy workflow itself, (made available through the export function of the GigaScience Galaxy instance), and the provenance statements about the inputs used. Everything in this RO, as well as the RO itself, is uniquely identified and can be referred to. This list of 5 rules was implemented as a checklist and whether an RO is compliant with this checklist can be automatically assessed using the RO quality assessment tool [51].

We observed some redundancy in capturing workflow inputs and outputs by the workflow-centric RO and the linkedISA conversion [38]. One could in fact envisage a reuse of the workflow description ontology, which is to enable a workflow-specific extension to the RO model, in an non-Basic Formal Ontology based linkedISA conversion of ISA-Tab document.

### ISA, NP and RO models: a complementary set of representational resources

We have described how these models have been harnessed to represent a computational experiment comparing genome assembler efficiency [20]. In order to convey the information payload held in each of the components more clearly, the SOAPdenovo2 case study website [52] includes a table summarising a number of query cases and highlights which model allows those queries to be answered. The overall study is described in an ISA-Tab document then converted to an RDF representation by the linkedISA software. At the other end of the spectrum, key experimental findings have been expressed as nanopublications, a semantic web compatible representation of the most salient results. All of these representations are placed in a broader context through a wrapper layer realised in the form of a Research Object.

## Discussion

The authors of the original SOAPdenovo2 paper [20] strived to make their work reproducible, making their source data, tools and scripts all accessible together with documentation. Yet, it still took about half a man-month worth of resources to reproduce the results reported from [20] in the Table 2 of their manuscript in Galaxy workflows. This indicates that it is not a lack of contributions or efforts by authors that hampers reproducible research practice. Rather, it is a lack of understanding of what needs to be provided to make this vision materialize. It also identifies a need to develop instructions for authors that go beyond traditional narrative papers. Our work revealed several distinct reasons leading to reproducibility collapse, even when software and data are available. These can be cast into the following categories:

1. ambiguities in resource identification,
2. absence of computer readable descriptions of inputs, computational workflows and outputs,
3. absence or limitation of available computational resources,
4. absence of identification of main elements in terms of experimental design such as predictor and response variables
5. limitation of depth and breadth of semantic artefacts, disambiguating unclear meaning of experimental elements.

So more generally, it is about dealing chiefly with ambiguity about experimental planning, followed by incompleteness in accounts.

Garijo *et al* [53] outlined some desiderata and guidelines to authors to improve the reproducibility of their results. Their paper focuses on reproducing computational results and the desiderata and guidelines emphasise on making available the input data, a data flow diagram, the software and its configurations together with intermediate data. The article we chose for this case study [20] does comply with most of the guidelines: the authors provided *bash* and *shell* scripts together with documentation and indications on how to obtain the input data. While intermediate data was not available, it could be obtained by running the given scripts. According to the classification provided by Garijo *et al* [53], the results could be reproduced by a novice user and by following the documentation, it was possible to reproduce the results with Galaxy workflows with minimum interaction with the authors. The GigaGalaxy platform now provides all the facilities, including workflow definitions and intermediate data, to re-enact the execution and reproduce the results. However, we identified other issues hampering reproducibility, which we describe later in this section.

But when considering the calls for reproducibility, let’s analyse its costs and who should bear them. *De novo* assembly of large genomes requires significant computational resources. Allowing for re-enacting those processes has an obvious economical footprint, which very rapidly places a cap on what can currently reasonably be offered. Typically, using an Amazon Web Services (AWS) instance ‘cr1.8xlarge’ with up to 244GB memory suited for repeating the large genome assembly, costs USD 3.5 per hour (this cost and subsequent calculations were done when the experiment was carried out). Repeating the YH genome assembly thus represents a USD 200-300 expenditure, excluding unavoidable storage cost. This raises the question *who most critically needs to reproduce all publication results?* Presumably, reviewers and journal editors should be the primary beneficiary of this attention. It is evident that not all results can be re-enacted owing to the associated operational costs, however, it is a pragmatic position to require that viable alternatives be provided to enable evaluation and review in order to establish trust in the results. This is the approach chosen by GigaScience.

The attention therefore shifts to certain qualitative aspects associated to the reporting of scientific experimentation. Despite the *big data* hype and associated controversial claims [54, 55], for most scientists, either computational or bench biologists, dispensing with the theory or with experimental designs is not an option. We show how to make the most of this information to perform a deeper review and help produce better reports.

The simplest issue to address when improving experimental reporting is resource identification. It constitutes our first and easiest recommendation: **unambiguously identify electronic resources, such as records downloaded from public repositories, by providing their official identifiers**. Typically, rely on a GenBank identifier instead of a possibly ambiguous sequence record name. This message is not only to authors, but also to reviewers and editors levering resources such as BioSharing [56], Identifiers.org and MIRIAM [57] repositories in this task. In line with our recommendation, **we propose that publishers provide a dedicated section for obligatory unambiguous references to electronic records**, similar to the traditional bibliographic reference section. This observation echoes recent findings about the lack of clear identification of materials and reagents in scientific papers [58] and recent amendments to data sharing policies by publishers such as PLOS [59].

A stronger recommendation would be to rely on Persistent identifiers (PID). PID such as DOIs or ORCID are meant to provide a stable addressing to digital objects, thus enabling unambiguous referencing for citation and access. On a more advanced level, persistent identifiers are enablers for data discovery and data reuse thanks to the resolution services which back them up and which hold the potential of realizing the “follow-your-nose” approach through data linking. Overall, they can be viewed as a key piece for interoperability [60]. There are yet to be pervasive and the practice of referencing accession numbers issued by well established databases is more widespread and is now supported by the Resource Identification Initiative and their RRID initiative [61].

The second recommendation is **to be explicit about experimental design and experimental variables, identifying the goal of the experiment, independent and response variables**. Table 2 of the present manuscript illustrates how variables and sample sizes could be reported in full, allowing a rapid assessment using a layout akin to a Wikipedia *info-box*. Interestingly, as the basic principles of experimental design remain irrespective of the field, it enabled ISA to be applied to non-biological experimental setups as in this case of algorithm comparison. Thinking in terms of experimental design identified a case of unbalanced factorial design, with study groups of unequal sizes since two bacterial genomes are used but only one genome of mid size and one of large size. Second, it lead one to ask about the state-of-the-art methods for evaluating algorithms to begin with [62, 63] and then for demonstrating process superiority [64]. In the absence of replication for several groups, the estimation of variance and standard deviation cannot be made. Owing to current compute costs, machine availability and project prioritisation, one may consider such a requirement excessive to demonstrate the performance of SOAPdenovo2 when a qualitative assessment may be deemed sufficient. It should, however, be pointed out that from a methodological point of view, applying principles of design of experiments would have certainly emphasised further and demonstrated more compellingly the benefits brought by SOAPdenovo2.

A complementary follow-up to the existing study could augment it by including additional genomes to collect more data points, thus ensuring replication and balancing of the design. For instance, the *Apis mellifera* (236Mb) genome could be used for the mid-size genome spot and dog (*Canis familiaris*, 2.4 gigabases of haploid genome) genome for the highest size spot. SOAPdenovo2 has been used to assemble the largest animal genome published to date (the 6.5GB *Locust* genome [65]). One could go further still; challenging SOAPdenovo2 and competitors with even larger plant genomes (also notorious for being highly repetitive).

Overall, this second recommendation offers a framework for critical appraisal. The authors conceded that, while the recommendation for testing for more data points along the slope to fully qualify the performance of SOAPdenovo2 algorithm could be justified, the reality of machine occupancy and incurred costs constitute obstacles to effective envelop testing. In addition, *de-novo* sequence assembly of genomes often requires specific parameter tuning to take the specifics of sequence libraries into account (e.g. bacterial artificial chromosome —BAC— or fosmid libraries). Still, those constraints need to be considered and discussed explicitly for the sake of clarity and exhaustivity when reporting results.

A scientific article is a narrative built on results collected through experimentation and facts uncovered through analysis. While the scientific endeavour demands neutrality towards facts, we all know too well the temptation to skew reports to highlight positive results. Hence, the next recommendation is **to remain neutral and report all findings of similar importance with the same weight**. Failing to do so can lead to *jumping to conclusions*, as we witnessed first hand when creating the NPs associated with the SOAPdenovo2 article based on the statements in the abstract.

Three assertions were initially generated: *(A1) increased genome coverage*, *(A2) decreased memory consumption*, *(A3) decreased run time*. Upon verification, *(A3)* turned out to be incorrect. While anecdotal, it is an actual example of *priming*, to use Tversky and Kahneman words [66], on the basis of the first two assertions. It also shows a benefit of the NP model, which requires reporting supporting facts back the claims, thus providing a proofing mechanism. Evidenc collected from Table 4 in the original manuscript [20] indicated that SOAPdenovo2 took slightly more time when compared to the other two algorithms to reach completion.

Our research results therefore reinforce the intuition that consistent and systematic reporting on the findings for each of the response variables defined in the experimental design needs to be made. In this instance, explicitly stating that SOAPdenovo2 software performs assembly task with significantly reduced *memory consumption*, with marginally increased *computation run time* and improved *genome coverage* provides a matter of fact assessment. This, in turns, would then be used to provide further comments. For instance that improvements to methods are often a matter of trade-offs and compromise. One may also consider identifying ahead of time which parameter gain is the most critical to the optimization task.

The observation also confirms the benefits of the declarative aspect of the ISA representation forcing to think in terms of experimental design, predictor and response variables, as well as the proofing aspect of the NP model.

Thus, the third recommendation can be further specified as **to report all findings corresponding to all the identified response variables**. For its ability to capture provenance and provide attribution, we chose the nanopublication model to report the main findings corresponding to the three response variables in the SOAPdenovo2 experiment, overcoming any priming issue.

Following this *model assisted review process*, which resulted in the identification of a small number of inaccuracies, the authors produced a correction article to officially communicate the amendment to their initial report [67]. Complementing this traditional approach, the release of nanopublications by the present work with the amended values highlights the model’s potential for disseminating evidence.

## Systems and Methods

### The ISA model

ISA is a Life-Science rooted general-purpose metadata tracking framework focused on supporting rich descriptions of the experimental conditions, with a growing community of users [14]. ISA model is at the core of a primary data repository of metabolomics datasets, for global and targeted metabolite profiling, including tracer-based pathway discovery experiments, now accounting for about 250 studies since it launched, 91 of which are currently public. Recent work with third party partners such as Biocrates AG and Bioplatform Australia resulted in the creation of ISA-tab based deposition pipelines, which should significantly increase the rate of deposition [68]. ISA is also central to secondary databases, such as the Stem Cell Commons, which focuses on serving highly curated functional genomics experimental evidence of processes determining cellular fate [69], or domain-orientated research repositories such as the Toxbank [70] and DiXA [71] projects, both centred on gathering biological signal by a variety of analytical techniques to monitor response to toxic chemical insult. Besides data repositories, scientific publishers have too selected ISA model for their data publication platforms: GigaScience and Scientific Data have validated the ISA model for its ability to accommodate consistently a wide range of experimental data. ISA-Tab is a hierarchical and tabular format designed to represent the experimental design, highlighting both predictor and response variables as well as considering replication of measurements, protocols, procedures and their parameters [72]. At its core is an underlying node-edge graph representation where node elements such as *materials* (a cell) and *data* (sequence) are input or outputs of *processes* (*e.g.* purification or data transformation). The ISA-Tab syntax supports the use of controlled terminologies and ontologies, tracking version and provenance information about those. The ISA open source software suite [42, 72–74] allows for the creation and manipulation of the ISA-Tab formatted information. For this work, the linkedISA software component was used to generate RDF statements from ISA-Tab formatted files, mapping the information to a semantic model and making explicit relations between the entities. In addition, the OntoMaton component [42] was employed to create nanopublications (see section 1), which were converted to RDF using NanoMaton [43].

### Galaxy workflow system

The Galaxy project aims to provide software infrastructure enabling scientists to execute complex computational workflows in the field of biology and sequence analysis. It is meant to support data analysis, but also to enable re-enactment and thus reproducibility. Galaxy is an open source, web-based application framework that benefits from a broad user base [16]. In addition to providing executable pipelines in a way that could support the reproduction of the original result, the framework is able to document the process of data analyses by providing a high-level overview diagram of the different analytical steps in the workflow, capturing versions of tools used in analyses and recording intermediate results. An instance of a Galaxy server was set up on GigaScience hardware and Galaxy workflows were defined for each of the algorithms tested.

### The Nanopublication model

The Nanopublication (NP) model is a mechanism for enabling the attribution of minimal biological assertions in a machine readable format [75]. Its main components are:

1. the assertion,
2. the provenance of the assertion,
3. the publication information of the NP itself, i.e. the attribution of the author(s) of the NP.

The recommended form for exchanging nano publications is by a Semantic Web implementation of the NP minimal model [76].

### The Research Object model

The Research Object model is an extendable, data aggregation model that is built upon a number of initiatives and community approaches. It is domain-neutral and enables to aggregate all information that is essential for understanding and reproducing an experiment result, associate supporting metadata along with them, and share them as a single, exchangeable object, i.e. a Research Object. The *researchobject.org* community involves scientists from a variety of domains to define a principled way for the identification, aggregation and exchange of scholarly information on the Web. It aims to identify the common principles underpinning these various existing solutions in order to create a harmonization of understanding and practices. The Research Object model [18] is one solution among these, providing an aggregation mechanism for components that are constituent parts of a broader research activity. Such components are interrelated with each other and are meant to provide the context to make research more effectively accessible and reusable.

The core RO model is lightweight and domain-neutral, simply providing a bundle structure for aggregating essential information that are needed for reproducing or reusing research results. In this paper, the science workflow-specific Research Object is used, which extends the core Research Object model with workflow-specific terminologies, like the definition of computational workflows, their steps, inputs and outputs data. To create the Research Object presented in this paper, the command-line RO Manager tool [77] was used, which offers the most flexibility for the range of annotations that we could provide. The resulting RO was published in the public RO repository and became accessible at the Research Object Portal [78] through a Permanent Uniform Resource Locator (PURL), RO PURL [79].

## Conclusion

Scientists are coming under increasing pressure from funding agencies to disseminate their research data and methods. In the life and biomedical sciences, community-standard repositories for storing such artefacts of research exist and are often mandated for use by journals. With basic metadata supplied, research outputs may also be assigned a Digital Object Identifier (DOI), a process overseen by DataCite [80], thus possibly facilitating discovery and citation. However, due to the complexity of todays research, making the results reported in publications in the biomedical sciences reproducible remains a major challenge. The task could however be facilitated by the use of virtual research environments (VRE), thanks to their data manipulation, editing and document hosting features they provide [81]. An example of a VRE is Galaxy, which has an emphasis on sequence data analysis and visualisation. Galaxy is able to facilitate collaborative science through the sharing of data and analytical workflows. The analyses reported by [20] in Table 2 of the initial manuscript were implemented in Galaxy in an attempt to replicate the results. If the products of the research lifecycle are managed by a VRE, one might imagine that it could also track the reproducibility of research using a combination of ISA, RO and NP. The reporting of scientific work can be greatly improved by taking advantage of the research objects reviewed in this work, namely, ISA, NP and RO data models in conjunction with Galaxy workflows to re-enact and validate data analyses. They present complementary features, which sweep the entire spectrum of the key points necessary to realise good digital preservation, from ISA and its emphasis on study plans, to the RO model dealing with computational workflow preservation and to NP, harnessed to structure and capture experimental conclusions. The strengths of these complementary data models lie in their respective philosophies. ISA and RO models both provide means to track experimental and computational workflows, with some level of acknowledged overlap, which is handled by deferring to the domain specific resources, with the RO project recommending ISA for the biological and life sciences domain.

Yet, it is unrealistic to expect researchers to be deeply acquainted with representation models and other semantic resources. Furthermore, not all computational optimitizations lend themselves to factorial analysis as performance tuning often involves platform specific elements, ranging from operating system dependencies, memory management issues and compiler levels. For such cases, new tools for packaging and platform virtualizations such as Docker [82] offer flexible and effective means to distribute computational tools and resources. To advance the role for data standards, models and computational workflows in scholarly publishing, further research is needed to make the process viable and above all, scalable. It is therefore critical to re-evaluate the existing tools supporting scholarly publishing. New tools are needed to help navigate and embed semantic representations by integrating representation models seamlessly, vocabulary servers for instance, possibly taking inspiration from NanoMaton [43], integrating Google collaborative spreadsheet environment with ontology lookup and tagging provided by OntoMaton and the NP model. Pivotal to this evolution are the interactions and community liaison needed among a variety of stakeholders, including vocabulary developers, service providers such as BioPortal [44], software developers and publishers, among others. Scholarly publishing has moved to a new phase and will continue to improve as new semantic artefacts are tested in a quest to enhance the article’s content or the discoverability and reuse of the underlying datasets. With peer review costing an estimated 2 billion US dollars each year, and criticisms that it is currently more of a faith rather than evidence-based process [83], the research work we report about constitutes an important foray into demonstrating how new principled methods can assist the review process, thus making it more accurate and quantitative. Publishers make the argument that they *add value* to the publication process, and these models offer unique potential to further the value proposition available to publishers in the capacity of providers of augmented content. In using the ISA, NP and RO models, we sought to meet requirements for sharing, reuse and repurposing, as well as interoperability and reproducibility. This fits with current trends to enhance reproducibility and transparency of science (e.g. [84–86]). Reproducibility in computational science has been defined as a spectrum [86], where a computational experiment that is described only by a publication is not seen as reproducible, while adding code, data, and finally the linked data and execution data will move the experiment towards full replication. Adhering to this definition, our RO-enabled computational experiment comes close to fulfilling the ultimate golden standard of full replication, but falls short because it has not been analyzed using independently collected data. The benefit offered by these models in terms of reproducibility is that it provides a context within which an evaluation of reproducibility can be performed. It does this by providing an enumerated and closed set of resources that are part of the experiment concerned, and by providing descriptive metadata (annotations) that may be specific to that context. This is not necessarily the complete solution to reproducible research, but at least an incremental step in that direction.

## Supporting Information

S1 Table List of resource name discrepancies. Summary of data file identification discrepancies found between the Galaxy instance set up at BGI and the original assemblathon/GAGE data used in manuscript by [20].
(PDF)

S2 Table The list of term requests submitted to OBI. The list of term requests submitted to OBI, the Ontology for Biomedical Investigation [40], to support the representation of findings by [20] as nanopublications.
(PDF)
