# Supplementary material for: From Peer-Reviewed to Peer-Reproduced in Scholarly Publishing: The Complementary Roles of Data Models and Workflows in Bioinformatics
Source: PLoS One. 2015 Jul 8;10(7):e0127612. doi: 10.1371/journal.pone.0127612 (PMC4495984; doi:10.1371/journal.pone.0127612)
Supplement: S1 Table — Summary of data file identification discrepancies found between the Galaxy instance set up at BGI and the original assemblathon/GAGE data used in manuscript by [20]. (PDF) [file pone.0127612.s001.pdf]

Table 1: List of resource name discrepancies.

Summary of data file identification discrepancies found between the Galaxy instance set up at BGI and the original assemblathon/GAGE data used in manuscript by [1].

Galaxy Server file names	original GAGE dataset file names
saureus_B_L4_37bp3500IS45X_2.fq	http://gage.cbcb.umd.edu/data/Staphylococcus_aureus/Data.original/frag_1.fastq.gz
saureus_B_L3_37bp3500IS45X_1.fq	http://gage.cbcb.umd.edu/data/Staphylococcus_aureus/Data.original/shortjump_1.fastq.gz
saureus_A_L2_101bp180IS45X_2.fq	http://gage.cbcb.umd.edu/data/Staphylococcus_aureus/Data.original/frag_2.fastq.gz
saureus_A_L1_101bp180IS45X_1.fq	http://gage.cbcb.umd.edu/data/Staphylococcus_aureus/Data.original/shortjump_2.fastq.gz

References

[1] Luo, *et al*. SOAPdenovo2: an empirically improved memory-efficient short-read de novo assembler. GigaScience. 2012 Dec;1(1):18. Available from: `http://dx.doi.org/10.1186/2047-217X-1-18`.
