# Supplementary material for: From Peer-Reviewed to Peer-Reproduced in Scholarly Publishing: The Complementary Roles of Data Models and Workflows in Bioinformatics
Source: PLoS One. 2015 Jul 8;10(7):e0127612. doi: 10.1371/journal.pone.0127612 (PMC4495984; doi:10.1371/journal.pone.0127612)
Supplement: S2 Table — The list of term requests submitted to OBI, the Ontology for Biomedical Investigation [40], to support the representation of findings by [20] as nanopublications. (PDF) [file pone.0127612.s002.pdf]

Table 1: The list of term requests submitted to OBI.

The list of term requests submitted to OBI, the Ontology for Biomedical Investigation [1], to support the representation of findings by [2] as nanopublications.

Class Label	Definition	Definition Source
Sequence assembly process	The process of merging and ordering shorter (read) fragments sampled from a set of larger sequences in order to reconstruct larger sequences	Assemblathon 1 [3]
Contig	A contiguous sequence of fragments	Sequence Ontology
Scaffold	A set of ordered and oriented contigs	Sequence Ontology
N50	N50 is a data item about a sequence assembly for evaluating assembly software performance. It provides a standard measure of assembly connectivity. The N50 of an assembly is a weighted median of the lengths of the sequences it contains, equal to the length of the longest sequence s, such that the sum of the lengths of sequences greater than or equal in length to s is greater than or equal to half the length of the genome being assembled.	N50 definition from Broad Institute & [3]
contig N50	N50 statistic computed for the contigs produced by the assembly process. A contig N50 is calculated by first ordering every contig by length from longest to shortest. Next, starting from the longest contig, the lengths of each contig are summed, until this running sum equals one-half of the total length of all contigs in the assembly. The contig N50 of the assembly is the length of the shortest contig in this list	[4]
scaffold N50	N50 statistic computed for the scaffold produced by the assembly process. The method for computing the value is similar to that of contig N50 but uses scaffold information instead of contig information	[4]
genome coverage	Total number of bases in reads, divided by genome size, assumed to be the reference size of 3.10 Gb for human and 2.73 Gb for mouse. Genome coverage, refers to the percentage of the genome that is contained in the assembly based on size estimates; these are usually based on cytological techniques. Genome coverage of 9095% is generally considered to be good, as most genomes contain a considerable fraction of repetitive regions that are difficult to sequence. So it is not a cause for concern if the genome coverage of an assembly is a bit less than 100%.	[4]
computation run time	the time expressed in second, minute, hour necessary for the program to complete a genome assembly. It is an important metrics as it indicates the resource occupancy	
maximum memory consumption	the maximum memory consumption is the peak use of computer memory required and necessary to complete a genome assembly. The metrics is a proxy for algorithm efficiency and parsimony	

References

[1] Brinkman, *et al*. Modeling biomedical experimental processes with OBI. J Biomed Semantics. 2010;1(Suppl 1):S7. Available from: http://dx.doi.org/10.1186/2041-1480-1-S1-S7.

[2] Luo, *et al*. SOAPdenovo2: an empirically improved memory-efficient short-read de novo assembler. GigaScience. 2012 Dec;1(1):18. Available from: http://dx.doi.org/10.1186/2047-217X-1-18.

[3] Earl, *et al*. Assemblathon 1: a competitive assessment of de novo short read assembly methods. Genome research. 2011 Dec;21(12):2224–2241.

[4] Yandell, Ence. A beginner's guide to eukaryotic genome annotation. Nat Rev Genet. 2012 May;13(5):329–342. Available from: http://dx.doi.org/10.1038/nrg3174.
